# Identification and Characterization of a Novel Allele of *Caenorhabditis elegans bbs-7*

**DOI:** 10.1371/journal.pone.0113737

**Published:** 2014-12-08

**Authors:** Kara Braunreiter, Shelby Hamlin, Jamie Lyman-Gingerich

**Affiliations:** Department of Biology, University of Wisconsin-Eau Claire, Phillips Hall 330, Eau Claire, Wisconsin, United States of America; Brown University/Harvard, United States of America

## Abstract

Primary cilia play a role in the sensation of and response to the surrounding environment. *Caenorhabditis elegans (C. elegans)* have primary cilia only on the distal tips of some dendrites. In order to better understand the relationship between receptor localization to cilia, cilia structure and cilia function, we have characterized a mutation originally identified in a forward genetic screen for mutants with defective PKD-2 ciliary localization. Through behavioral assays and examination of the structure of cilia in the *cil-5* (*my13)* mutant animals, we have found that *my13* disrupts not only receptor localization, but also some cilia-mediated sensory behaviors and cilia structural integrity. We have identified the *my13* lesion and found that it is a missense mutation in *bbs-7*, an ortholog of human BBS-7, a gene known to affect human cilia and to be involved in Bardet-Biedl syndrome. Finally, we show that *bbs-7(my13)* also affects the glia cells which support the cilia.

## Introduction

Primary cilia play a role in sensation of and response to the surrounding environment. They do so for many different cell types. For example, in humans, cilia on kidney epithelial cells act as flow sensors for urine, cilia on ectodermal and mesenchymal cells in the limb bud are required for proper patterning, and cilia on the rod and cone photoreceptor cells are required for maintenance and function of these cells [1]–[3]. The structure of primary cilia and the process by which they are formed are well-conserved across multiple cell types and organisms. The cilium protrudes from the cell membrane and contains a 9+0 arrangement of microtubule doublets. All proteins necessary for the form and function of the cilium must be transported into and within the cilium as there is no translation within the structure. A combination of molecular motors and cargo-associated proteins actively transport cargo within the cilium in a process known as intraflagellar transport (IFT) [4], [5].

While the general structure of primary cilia is conserved between cell types, the cell-type related function of each cilium is mediated by the localization of specific molecules to that cilium. For example, in humans, polycystin-1 (PC1) and polycystin-2 (PC2) localize to primary cilia of renal tissue where they interact to form a calcium channel and when mutated, cause autosomal dominant polycystic kidney disease [6], [7]. The dysfunctional cilia result in the inability of epithelial cells to sense fluid flow through the lumen and lead to abnormal cell proliferation and, ultimately, cyst formation [8]. Whereas mutations in genes encoding proteins specific to certain cilia produce cell-type specific effects, mutation of genes involved in cilia structure and general cilia function results in pleiotropy due to defects in a wide range of tissue types [9]. For example, Bardet-Biedl (BBS), Meckel-Gruber (MKS), and Joubert Syndrome (JBTS) are all classified as ciliopathies because the causative genes function in cilium development in an array of tissue types. These ciliopathies share many phenotypes including renal cysts, polydactyly and *situs inversus* [2], [10]–[13].

The complex phenotypes associated with cilia disorders in vertebrates, and the embryonic lethality resulting from lack of primary cilia make the use of an alternate model attractive to study cilia structure and function. Basic morphology and signaling molecules of the cilium are well-conserved across metazoans [14], meaning that findings about cilia structure and function in one organism, such as *C. elegans*, can be applicable to other organisms. Of the 959 cells in the *C. elegans* adult hermaphrodite, only 60 cells, a subset of the neurons, contain primary cilia. The *C. elegans* male has an additional 87 neurons, of which 48 have been shown to be ciliated. Because of the relatively small number of ciliated cells in the worm, *C. elegans* are viable without functional cilia making it possible to study the effects of cilia loss in a living organism. Nevertheless, cilia are important to the worm as ciliated neurons are involved in sensory behaviors such as chemosensation, osmosensation, thermosensation and mate finding [15]. (For a comprehensive introduction to primary cilia in *C. elegans*, the reader is directed to the review by Inglis and colleagues [16].) Thus, *C. elegans* provide a tractable model system for the analysis of primary cilia assembly and function.

Examples of cilia-related genes with orthologs in both *C. elegans* and humans include PKD-2 (*polycystic kidney disease-2)*/PC2 (polycystin 2) and LOV-1(*location of vulva defective-1)*/PC1 (polycystin 1). *C. elegans* PKD-2 interacts with LOV-1 to form a calcium channel in the cilia membrane of male-specific neurons [15]. The human orthologs, PC1 and PC2, localize similarly to cilia in the human renal epithelium. When PKD-2/PC2 is not properly localized to the ciliary membrane, defects in male mating behavior occur in *C. elegans* [17]–[19] and cyst formation occurs in humans [20].

One question that can be addressed using *C. elegans* is how PKD-2 is transported from the golgi body to the ciliary base and then localized on the ciliary membrane. Transport of PKD-2 within vesicles has been proposed for movement to the ciliary base [21]. However, unlike some other receptor proteins, such as OSM-9 and OCR-2, PKD-2 is not localized to the ciliary membrane by intraflagellar transport and the mechanism by which this transport occurs is unclear [21], [22]. A forward genetic screen to identify additional factors involved in PKD-2 ciliary localization (the Cil phenotype) [17] isolated *cil-5(my13)*. Cilia of male-specific neurons of *C. elegans* homozygous for *my13* curve slightly inward and accumulate additional PKD-2::GFP at the ciliary base [17].

We present further characterization and cloning of *cil-5(my13)*, a novel allele of *bbs-7*. Here, we show that *bbs-7*(*my13)* mutant animals exhibit specific chemosensory defects and aberrations in cilia structure which do not overlap entirely with phenotypes of previously reported *bbs-7* alleles. In addition, although the BBS genes are expressed exclusively in ciliated neurons [23], we observed changes in glial cells in the *bbs-7(my13)* mutant animals. These results provide additional evidence for the role of *bbs-7* in cilia structure and cilia function, and crosstalk within the sensilla between neurons and glial cells.

## Materials and Methods

### Worm Strains

The following worm strains were used: JLG3 *(him-5(e1490)V; PKD-2::GFP I; bbs-7(my13) III),* JLG5 *(him-5(e1490)V; bbs-7(my13) III)*, CB1490* (him-5(e1490)V)*; CB4856; CHB28 *(oyIs45 [odr-1pro: YFP, lin-15(+)V]; nsEx1153 nsEx2073 [F16F9.3pro: mCherry, itr-1*pro: CFP + pRF4]), JLG23* (bbs-7(m13) III*; *oyIs45 [odr-1pro*: YFP, *lin-15(+)]*V; *nsEx1153 nsEx2073 [F16F9.3*pro: mCherry, *itr-1*pro: CFP + pRF4]); MT3645 *(osm-12(n1606)III)*; RB1268 *(osm-12(ok1351)III)*; ZP634 (*jhuEx634 [Parl-13::bbs-7::GFP+pRF4])*.

### SNP Mapping and Genome Sequencing

*bbs-7(my13)* homozygous mutant animals carrying PKD-2::GFP were crossed to the polymorphic Hawaiian strain, CB4856. F1 heterozygotes were identified by the presence of PKD-2::GFP and allowed to self-fertilize. F2 homozygous mutant animals were identified by dye-filling (tailDyf), allowed to self-fertilize, and the F3 progeny were pooled. Genomic DNA isolation was performed using the Gentra Puregene kit (Qiagen) as described in Doitsidou et al. 2010 [24]. Genomic DNA samples were also prepared using CB4856 and JLG3 strains as controls. Library preparation and whole genome sequencing were performed at the University of Wisconsin Biotechnology Center. Analysis of the Hawaiian/Bristol ratios of SNP loci was done using Next Generation EMS Mutation Mapping (http://bar.utoronto.ca/ngm/).

### Dye-filling

Dye filling was performed as described [25] with the following modifications. For the assay (dye concentration 25 µg/mL), worms were exposed to the dye for two hours. Dye-filling was assessed by observing the extent of dye-filling in the amphids (nose tip, dendrites and cell bodies) and the phasmids.

### Epifluorescence Microscopic Analysis

Animals were mounted as detailed in [17]. Fluorescence analysis was performed on a Nikon i80 using a 60x (NA1.4) oil objective. Images are either of single focal planes or optical sections projected as Z-series that were manipulated using NIS elements software. The imaging of the animals with F16F9.3pro: mCherry was performed on a confocal microscope at the model core of the Mayo Translational PKD Center.

### Chemotaxis Assays

60mm plates containing nematode growth medium were divided into 5 sections of equal width (labeled A–E). 2 µl of the test chemical were placed at one end of the plate (section A) and 2 µl of the diluent (ethanol) were placed at the other end of the plate (section E). After rinsing age-synchronized 1 day old adult worms in M9, approximately 50 worms were placed in the center of the plate, and their movements were tracked by counting the number of animals in each section every 10 minutes for an hour. The worm chemotaxis index (WCI) was calculated using the equation: WCI  =  [(A+B) – (D+E)]/(A+B+D+E). In order to measure whether there was any difference in the degree of attraction between the strains, we calculated the percentage of animals in the section closest to the chemical compared to the number of total animals that approached the chemical: this is reported as percent strong attraction. (Strength of avoidance was similarly calculated by observing the number of animals in the section furthest from the chemical.)

### Quantification of Vacuoles

Animals of each strain were age-matched by bleaching gravid hermaphrodites to obtain synchronous clutches of embryos. The presence of vacuoles in the sheath cells of CHB28 and JLG23 animals was assessed by looking for round structures that were devoid of *F16F9.3* pro: mCherry expression in the sheath cells. Each animal was scored as either having vacuoles or not having vacuoles. In each experiment, between five and fifteen animals per age per strain were assessed. The experiment was repeated four times, and the aggregate data are presented.

## Results

### *bbs-7* is required for amphid, phasmid and CEM neuron structure

**Figure 1:**
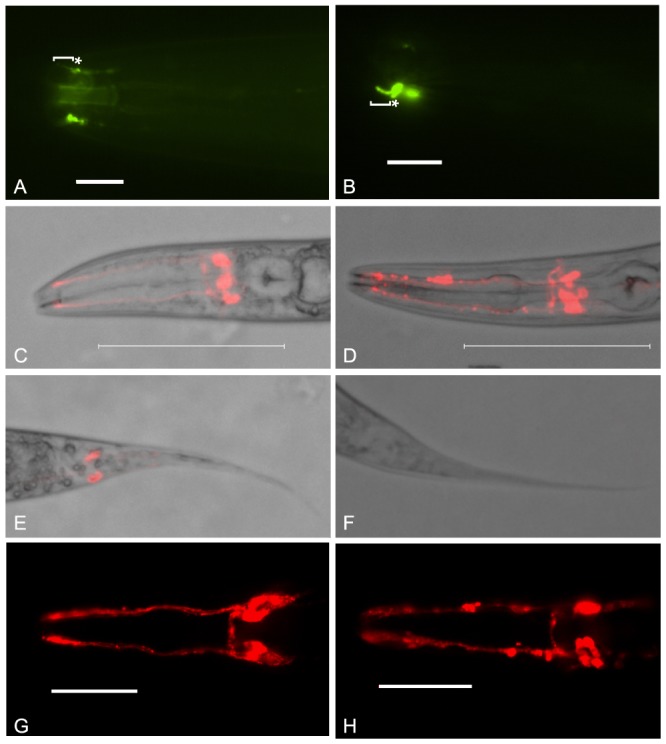
*bbs-7(my13)* animals exhibit defects in PKD-2::GFP localization and the ability of sensory neurons to take up lipophilic dye. PKD-2::GFP localizes to the cilium proper and base of the male-specific CEM neurons in wild-type worms (bracket and asterisk, respectively, in A). The *bbs-7(my13)* male CEM neurons also have PKD-2::GFP in the cilium proper and base but the CEM cilia curve inward and there is additional accumulation of PKD-2::GFP in the base (bracket and asterisk, respectively, in B). Wild-type worms take up DiI in both the dendrites and cell bodies of the amphids (C and G) and the phasmids (D). *bbs-7(my13)* animals take up dye in the amphid neurons (D and H) but not the phasmid neurons (F) and the quality of the dye-filling in the amphid neurons is not equal to wild-type. Different worms are shown in panels C and G, and D and H. Scale  = 100 microns in A and B. Scale  = 10 microns in E and F. Anterior to left.

*C. elegans* homozygous for *bbs-7(my13)* exhibit defects in male-specific ciliated neurons and some ciliated neurons common to both males and hermaphrodites. As previously reported, PKD-2::GFP localization in the male-specific CEM neurons is aberrant [17]. The CEM cilia of mutant animals curve inward instead of extending straight toward the nose tip indicating possible structural defects in the cilia. There is also increased accumulation of PKD-2::GFP at the ciliary base in the mutant animals compared to wild-type (fig. 1 A and B). Both males and hermaphrodites contain amphid and phasmid neurons. A subset of wild-type amphid and phasmid neurons are exposed to the environment and the lipophilic fluorescent dye, DiI, labels these neurons. The phasmids of *my13* mutant hermaphrodites fail to take up DiI (the tailDyf phenotype) while the amphids fill with DiI (fig. 1 C–H and[17]). However, the amphid dye-filling phenotype is qualitatively different in *bbs-7(my13)* mutant animals compared to wild-type. In wild-type animals, the amphid dendrites fill uniformly with dye, but in mutant animals, labeling is not consistent and the dye appears to accumulate in pockets along the length of the dendrites (fig. 1). Both the phasmid and the amphid dye-filling defects are completely penetrant.

### *bbs-7(my13)* modulates behavioral responses to sensory cues

Defects in the mating behavior of *bbs-7(my13)* males, which is regulated in part by the CEM neurons, have been reported [16]. Because of the dye-filling defects observed in the *bbs-7(my13)* animals, we asked if behaviors attributed to the function of other ciliated neurons were affected. We tested the responses of the *bbs-7(my13)* mutant worms to diacetyl, benzaldehyde and isoamyl alcohol because responses to these chemicals are mediated by different subsets of ciliated neurons.

**Figure 2:**
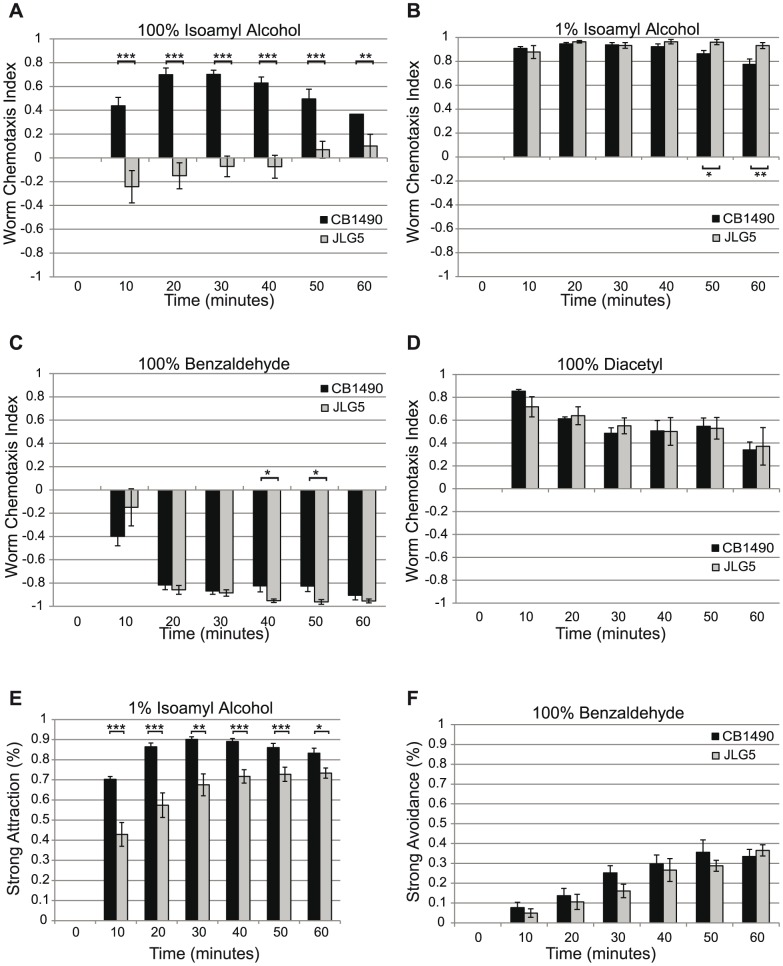
*bbs-7(my13)* animals exhibit altered responses to some volatile chemicals. Wild-type worms approach a point source of 100% isoamyl alcohol more than *bbs-7(my13)* mutant animals (A). Wild-type and *bbs-7(my13)* mutant animals respond in a similar fashion to 1% isoamyl alcohol, 100% benzaldehyde and 100% diacetyl (B, C and D) when assessed using the Worm Chemotaxis Index. *bbs-7(my13)* animals do not show the same strength of attraction as wild-type animals to 1% isoamyl alcohol (E) but show the same degree of avoidance of 100% benzaldehyde (F). Error bars indicate standard error of the mean. P-values calculated using a standard t-test. ***<.001 **<.005 *<.05

Response to diacetyl is mediated in part by the AWA neurons [26]. Attraction to isoamyl alcohol and low concentrations of benzaldehyde is mediated by the AWC neurons while avoidance of higher concentrations of benzladehyde requires the AWB neurons [26]. *bbs-7(my13)* animals responded similarly to wild-type animals to both high (100%) and low (1%) concentrations of benzaldehyde and diacetyl (fig. 2 and data not shown). However, the mutant animals' response to isoamyl alcohol was concentration dependent: while wild-type animals were attracted to both 100% and 1% isoamyl alcohol, *bbs-7(my13)* animals avoided or showed little attraction to 100% isoamyl alcohol (fig. 2). Although *bbs-7(my13)* animals showed a similar attraction to 1% isoamyl alcohol compared to wild-type animals as measured by the Worm Chemotaxis Index, we observed that the mutant animals did not approach the point source of the chemical as closely as the wild-type animals. For 1% isoamyl alcohol, specifically, there was a significant difference in attraction strength for each time point tested. The chemotaxis data suggest that some but not all ciliated neuron functions are affected in *bbs-7(my13)* animals. The different responses observed for the wild-type and mutant strains could be a function of specific defects in ciliated neurons and receptor localization or due to reduced mobility of the *bbs-7(my13)* mutant animals. To examine this second possibility, we tested both the roaming ability of individual animals on food and the response of the animals in a chemotaxis assay where the plate contained only diluent. There is no significant difference in the area covered by the *bbs-7(my13)* mutant animals as compared to wild-type animals when the individual animals are on food (data not shown). However, in the absence of odorant or food on the plate, *bbs-7(my13)* mutant animals do not travel as far as wild-type animals (table 1). When exposed to a strongly attractive (1% diacetyl) or strongly repulsive (100% benzaldehyde) odorant, *bbs-7(my13)* mutant animals display an equal or greater ability to move toward or away from the odorant compared to wild-type animals (table 1). Therefore, the differences seen in response to the chemicals are unlikely to be due to a difference in locomotion ability.

**Table 1:** Distance traveled in response to different odorants.

Strain	Point sources	Point sources	Point sources
	Ethanol (A and E)	100% Benzaldehyde (A) and Ethanol (E)	1% Diacetyl (A) and Ethanol (E)
*him-5(e1490)*			
10 minutes	0.134 (±0.084)	0.0317 (±0.0372)	0.504 (±0.235)
20 minutes	0.210 (±0.094)	0.0944 (±0.0790)	0.651(±0.178)
30 minutes	0.246 (±0.113)	0.164 (±0.0683)	0.718 (±0.120)
*bbs-7(my13)*			
10 minutes	0.0153 (±0.0197)	0.0126 (±0.0142)	0.564 (±0.188)
20 minutes	0.0480 (±0.0419)	0.0604 (±0.0832)	0.734 (±0.137)
30 minutes	0.108 (±0.0615)	0.101 (0.0799)	0.813 (±0.0657)

Percentage of animals in the sections closest to the point sources of chemicals or diluent (A and E) in the chemotaxis assays. Wild-type animals traveled further on the plate than *bbs-7(my13)* animals when only ethanol, the diluent, was present on the plate (p-values: at 10 minutes  = 0.00158, at 20minutes  = 0.000525, at 30minutes  = 0.00873). In the presence of a strong repellent (100% benzaldehyde) or a strong attractant (1% diacetyl), both wild-type and *bbs-7(my13)* animals travel similar distances on the assay plate (p-values for 100% benzaldehyde: at 10 minutes  = 0.150, at 20 minutes  = 0.375, at 30 minutes  = 0.08501; p-values for 1% Diacetyl: at 10 minutes  = 0.589, at 20 minutes  = 0.322, at 30 minutes  = 0.0681). Value in parentheses indicates standard error of the mean.

### *bbs-7(my13)* affects glial cell morphology

**Figure 3:**
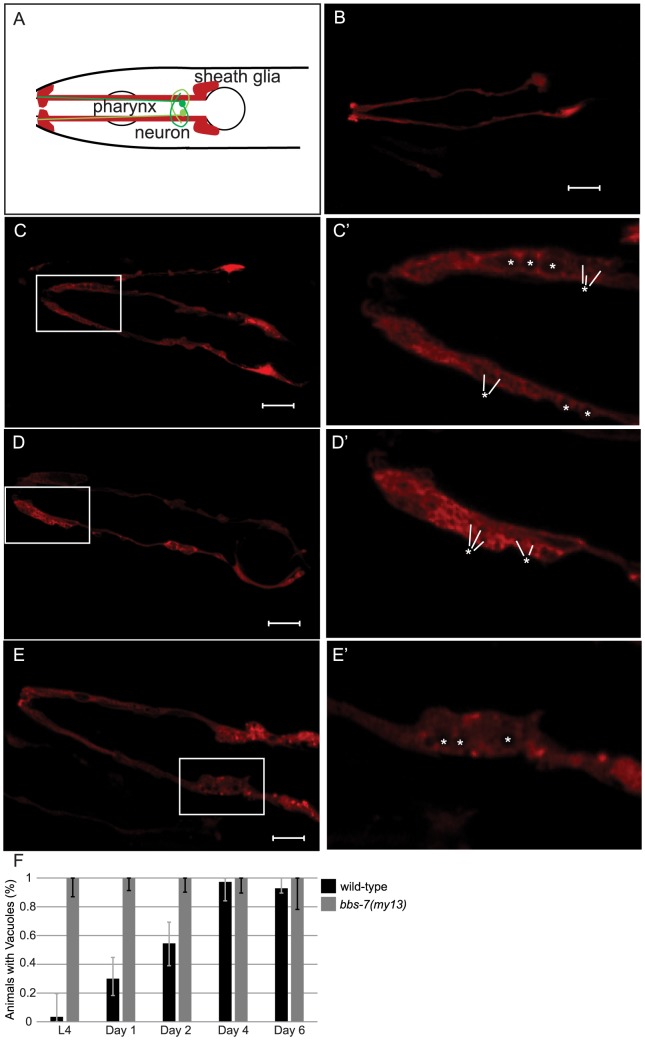
*bbs-7(my13)* animals have vacuoles present in amphid sheath cells. The sheath glia surround the amphid neurons (A). Wild-type worms express F16F9.3pro: mCherry in the amphid sheath cells (B). *bbs-7(my13)* also express F16F9.3pro: mCherry in the amphid sheath cells but the sheath cells have round areas which lack expression (C, D and E). Enlarged views of boxed areas in C, D and E are shown in C′, D′, and E′, respectively. (Asterisks indicate vacuoles). Scale  = 20 microns. Anterior to left. The percent of animals with vacuoles in the amphid sheath cells differs between wild-type and mutant animals at the L4, day 1 adult, and day 2 adult stages but not at the day 4 adult or day 6 adult stage (F). (Bars indicate the 95% confidence interval calculated using a 1-sample proportions test with continuity correction.)

The dendrites of the twelve neurons in each of the amphids are associated with two glial cells, the sheath cell and the socket cell [27], [28]. The presence of matrix-filled vesicles in cilia mutants has previously been reported [25]. Similarly, we observed that the sheath cells of *bbs-7(my13)* mutant animals contain vacuoles in the L4 and adult stages (fig. 3). By contrast, L4 wild-type animals have no visible vacuoles within their sheath cells, but, as the animals age, small vacuoles begin to build-up within the sheath cells. The vacuoles in the adult mutant animals appear larger and more numerous than those in the wild-type animals. The percentage of *bbs-7(my13)* animals with vacuoles in the amphid sheath cells approaches 100% at both L4 and adult stages (fig. 3). Thus, it appears that the mutant animals possibly have a defect in the sheath cells.

### *my13* is an allele of *bbs-7*, a component of the BBSome

**Figure 4:**
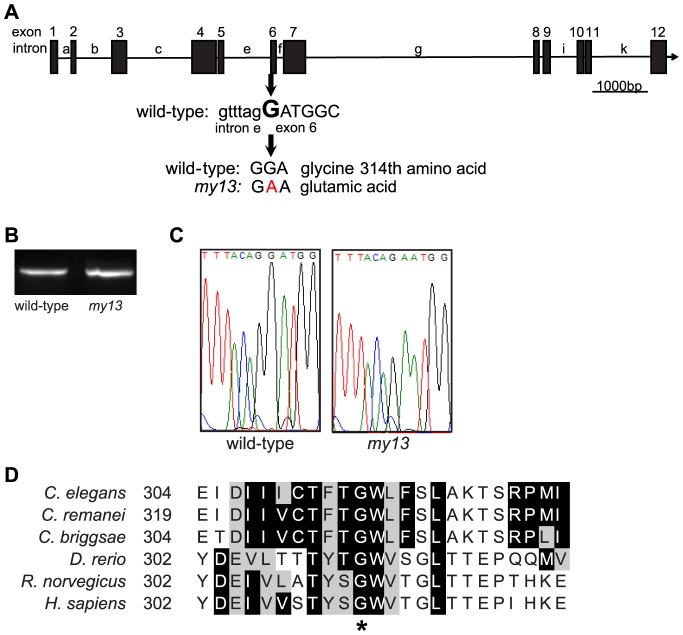
The *my13* mutation affects *bbs-7*. Structure of the *bbs-7* gene (A). Exons are numbered and introns are lettered. The *my13* mutation changes the first nucleotide of exon 6 from a G to an A. RT-PCR of mRNA isolated from wild-type and homozygous *my13* animals results in products that are the same size (B). Chromatograms showing partial wild-type and mutant *bbs-7* sequence (C). Alignment of the region of BBS-7 affected by the mutation in *my13* (D). Alignments of BBS-7 proteins from six species were generated in ClustalOmega and displayed with Boxshade using a threshold of 50% sequence identity. Conserved and similar amino acids are shown in black and gray boxes, respectively. Asterisk denotes the *C. elegans* glycine (amino acid 314) affected by the *my13* mutation.

The *my13* allele was originally isolated because of the PKD-2::GFP mislocalization phenotype exhibited by homozygous mutant animals [17]. In order to identify the residue affected by the *my13* mutation, we undertook a whole genome sequencing and single nucleotide polymorphism mapping approach [24]. We identified a non-synonymous SNP in *bbs-7*, the ortholog of human BBS7 (fig. 4A). Because the *my13* mutation results in a substitution of an adenine for a guanine at the first nucleotide of exon 6, we reasoned that *my13* might either result in aberrant splicing of the *osm-12* transcript or substitution of glutamic acid for glycine at position 314 in the protein. Amplification of *osm-12* mRNA from both wild-type and homozygous mutant animals resulted in products that were the same size (fig. 4B); sequencing of these products confirmed that only the identity of a single nucleotide differed between them (fig. 4C). Because the transcripts are the same length and there are only single peaks on the chromatogram, the *my13* mutation likely does not affect splicing but instead changes the amino acid sequence of the translated protein. However, we cannot rule out the possibility that some transcripts are mis-spliced and that these affect the mutant phenotype.

There are three previously reported alleles of *bbs-7* (also referred to as *osm-12*) in *C. elegans*. We examined the phenotypes of two of these. *osm-12(n1606)* results in a nonsense mutation at nucleotide 2899 and is likely a null allele [12] while *osm-12(ok1351)* results in a loss of exons 6 and 7 and is also likely null [29]. Both *osm-12(n1606)* and *osm-12(ok1351)* mutant animals are completely dye-filling defective; they fail to take up any dye in either the amphid or phasmid neurons ([12], [29] and our own data). This phenotype differs from that observed in the *bbs-7(my13)* mutant animals, which we report to be only partially dye-filling defective. This means that the *my13* allele retains function to permit dye-filling in the amphid neurons suggesting a functional distinction between *my13* and the other alleles.

Complementation analysis revealed a complex relationship between the *my13*, *n1606*, and *ok1351* alleles, depending on the phenotype evaluated. When observing the dye-filling phenotype of the trans-heterozygotes for any pairwise combination of the three alleles, the weaker mutant phenotype predominates: all animals are dye-filling defective in the phasmids and exhibit abnormal dye-filling of the amphids (table 2). The PKD-2::GFP localization phenotype exhibits partial complementation when examining the *my13/n1606* and the *my13/ok1351* trans-heterozygotes: 38% and 44% of animals, respectively, have increased accumulation at the ciliary base accompanied by inward curvature of the CEM cilia (table 2). This partial complementation may indicate that, unlike *n1606* and *ok1351*, *my13* does not result in complete loss of function of *bbs-7.*

**Table 2:** Complementation tests involving three alleles of *bbs-7* reveal complexity in interactions between alleles.

	my13	ok1351	n1606
*my13*	90% tailDyf and 10% Dyf (n = 50); 89% Cil (n = 18)	97% tailDyf and 3% Dyf (n = 79); 38% Cil (n = 55)	87% tailDyf and 13% Dyf (n = 60); 44% Cil (n = 34)
*ok1351*		14% tailDyf and 86% Dyf (n = 29)	11% tailDyf and 89% Dyf (n = 27)
*n1606*			3% tailDyf and 97% Dyf (n = 36)

Animals homozygous for the *my13* allele do not take up fluorescent dye in their phasmids and exhibit abnormal dye-filling in the amphids (tailDyf) while *ok1351* homozygotes and *n1606* homozygotes do not take up dye in either the amphids or phasmids (Dyf). Trans-heterozygotes involving the *my13* allele (*my13/ok1351* and *my13/n1606)* have a similar phenotype to the *my13* homozygotes. *ok1351/n1606* trans-heterozygotes are pre-dominantly dye-filling defective. Wild-type animals (*him-5(e1490))* were 100% nonDyf (n = 46). The percentage of animals with PKD-2::GFP mislocalization (the Cil phenotype) is less in the trans-heterozygotes compared to animals homozygous for *my13* allele.

We examined whether expression of full-length wild-type BBS-7 in *bbs-7(my13)* mutant animals could rescue the phasmid dye-filling defects. Overexpression of BBS-7: GFP in a wild-type background itself results in phasmid dye-filling defects some of the time (table 3). This suggests that the level of BBS-7 expression in the phasmids may be important. While expression of BBS-7::GFP in the *bbs-7(my13)* mutant background does not completely rescue the phasmid dye-filling defects, the percentage of animals that fill normally with dye approaches wild-type levels (75% versus 79% nonDyf).

**Table 3:** BBS-7::GFP restores phasmid dye-filling in *bbs-7(my13)* mutant animals.

Strain	Ability to dye-fill (%)
*him-5(e1490)*	100% nonDyf (n = 62)
*bbs-7(my13)*	100% tailDyf (n = 64)
BBS-7::GFP	79% nonDyf and 21% tailDyf (n = 62)
*bbs-7(my13)* with BBS-7::GFP	75% nonDyf and 25% tailDyf (n = 56)

Animals homozygous for the *my13* allele do not take up fluorescent dye in their phasmids and exhibit abnormal dye-filling in the amphids (tailDyf) while wild-type (*him-5(e1490)* animals take up dye in both the amphids and phasmids. Some wild-type and *bbs-7(my13)* animals carrying the BBS-7::GFP extrachromosomal array are tailDyf.

## Discussion

BBS-7 is one of a family of genes known to cause Bardet-Biedl Syndrome, a pleiotropic ciliopathy, in humans [30]. BBS7 forms a complex, known as the BBSome, with six other BBS proteins. Components of the BBSome have been shown to play key roles in the function of primary cilia in both humans and *C. elegans*: the BBSome complex is necessary for vesicle trafficking to the ciliary membrane, proper intraflagellar transport and, specifically, turnaround of the transport mechanism at the tip of the cilium [31]–[33]. BBS proteins also play roles in assembly of the intraflagellar transport complex necessary for movement of molecules within the cilium [12], [34], neuropeptide secretion [35], and gene expression regulation [36]. Dysfunction of the BBS proteins leads to Bardet-Biedl syndrome in humans and sensory defects in *C. elegans.* Here, we characterize and clone *my13*, a novel allele of *bbs-7* and provide additional evidence for the role of *bbs-7* in primary cilia structure, sensory neuron function, and communication between neurons and sheath glia.

### *my13* affects a conserved residue of *bbs-7*

We show that *my13* results in the substitution of an adenine for a guanine at the first nucleotide of exon 6, which would likely lead to glutamic acid instead of glycine at position 314 in the BBS7 protein. This glycine residue is highly conserved among both the *Caenorhabditis* genus and vertebrates, indicating possible functional significance. The affected residue also falls in a 252-amino acid region of human BBS7 that is highly conserved between human BBS7 and human BBS2 as well as BBS2 in other species including *C. elegans* [30]. This common region is predicted to encode a six-bladed β-propeller structure that has been suggested to mediate interactions of BBS7 with other proteins within the BBSome and the chaperonin complex [30], [37]. The glycine affected by *my13* is predicted to be located in the coil or turn between two β-strands [38], and we postulate that the change from a small hydrophobic glycine to a large negatively-charged glutamic acid may have an effect on the conformation of the BBS7 protein.

The partial dye-filling defect of *bbs-7(my13)* animals combined with the altered cilia structure observed when examining ciliary receptor localization suggest that *bbs-7* is important for primary cilia structure. In BBS-7 and BBS-8 null worms, the IFTA and IFTB complexes dissociate for anterograde transport [32], and interactions between BBS1, BBS7 and BBS9 are disrupted [33]. In mice, BBS7 has been shown to be a component of both the BBSome and the BBS chaperonin complex [39]. BBS6, BBS10, and BBS12 form a complex with the CCT chaperonins in vertebrates, and it has been proposed that these proteins mediate the interactions of CCT chaperonins with BBS2 and BBS7 [40]. It will be interesting to determine whether the *my13* mutation results in disruption of the interactions between BBSome components or, alternatively, of the BBSome with the CCT chaperonins.

### *bbs-7(my13)* affects functions of specific neurons

In nature, *C. elegans* are soil-dwelling and depend on a relatively simple nervous system of 302 neurons to identify, integrate and respond to the complex array of chemicals present in order to find food and avoid toxins. Distinct subsets of neurons mediate responses to aversive and attractive odors: the AWA and AWC neurons detect attractive odors while the ASH, ADL, AWB and ASK neurons detect aversive odors [26]. *bbs-7(my13)* animals do not respond like wild-type animals to the volatile odorant isoamyl alcohol, but respond normally to benzaldehyde and diacetyl. The different behavioral responses to the volatile odorants may be due to altered overall perception of specific chemicals or an inability to finely map changing concentrations of the chemicals over time. The phasmid neurons have been shown to be chemosensory and integral for the avoidance of the chemical SDS [41]. It is possible that the chemosensory and phasmid dye-filling defects observed in the *bbs-7(my13)* animals are interrelated.

### Neuron-sheath cell interactions are affected in *bbs-7(my13)* mutant animals

*bbs-7* is expressed specifically in ciliated neurons in *C. elegans* [12], [32]. Thus, we would expect to see mutant phenotypes involving the ciliated neurons. We also observe accumulation of vacuoles in the supporting sheath glial cells in *bbs-7(my13)* mutant animals. These glial cells have been associated with the remodeling of neuronal endings [42] and communication between neurons and glial cells is important for function of both [43]. This primary defect in ciliated neurons resulting in a secondary defect in sheath glial cells is not unique: a similar phenotype has been observed in *daf-19* mutants [44]. A number of intraflagellar transport (IFT) and other cilia mutants also have accompanying defects in sheath cell structure including *che-11, che-10, osm-3, che-12*, and *mec-8*. Mutations in each of these genes are associated with accumulation of vesicles in the amphid sheath cells [25]. More specifically, these matrix-filled vesicles are indicative of defects in vesicle release. While we have not identified the *my13* vacuoles as vesicles, the phenotype that we observe would be consistent with the vesicle accumulation previously reported for other mutants. Proper amphid glia formation has been shown to be required for cilia formation and function of some of the amphid neurons [28]. Thus, the mutant phenotypes observed in *bbs-7(my13)* animals are likely to be the result of both cilia dysfunction itself and perturbation of cilia-sheath cell interactions.

The identification of a novel allele of *bbs-7* provides additional evidence of BBS7 function in cilia structure, sensory function, and interaction between ciliated neurons and glial cells. Future characterization of the nature of the protein encoded by *bbs-7(my13)* may be useful in providing details about the interactions between BBS7 and other components of the BBSome as well as between BBS7 and the chaperonin complex.
